# Epigenome-Wide Association Study of Depressive Symptoms in Black Women in the InterGEN Study

**DOI:** 10.3390/ijms25147681

**Published:** 2024-07-12

**Authors:** Brittany Taylor, Yihong Zhao, Nicole B. Perez, Stephanie Potts-Thompson, Cindy Crusto, Ruth Masterson Creber, Jacquelyn Y. Taylor

**Affiliations:** 1 School of Nursing, Columbia University, New York, NY 10032, USA; bt2542@cumc.columbia.edu (B.T.); yz2135@cumc.columbia.edu (Y.Z.); sp3879@cumc.columbia.edu (S.P.-T.); rm3284@cumc.columbia.edu (R.M.C.); 2 Rory Meyers College of Nursing, New York University, New York, NY 10010, USA; nbp273@nyu.edu; 3 School of Medicine, Yale University, New Haven, CT 06510, USA; cindy.crusto@yale.edu

**Keywords:** epigenetics, depression, mental health, Black women, women’s health

## Abstract

(1) The prevalence of depression is two times higher in women than men. Black women have an increased risk of depression due to stressors such as low socioeconomic status and perceived discrimination. Depression is likely influenced by both genetic and environmental factors. Psychosocial stressors can influence DNA methylation (DNAm), leading to changes in gene expression and ultimately, depression. The objective of this study was to examine associations between DNAm and depressive symptoms in Black women. (2) This study was a secondary analysis of data from the Intergenerational Impact of Genetic and Psychological Factors on Blood Pressure (InterGEN) Study. Perceived discrimination was assessed using Krieger’s Experiences of Discrimination and Waelde’s Race-Related Events Scale, and participants were screened for depressive symptoms with the Beck Depression Inventory. Raw data from saliva samples were analyzed using the Illumina Infinium Epic (850 K) BeadChip and then preprocessed in RStudio. (3) Differential methylation analysis identified DNAm sites and regions associated with depressive symptoms. Six DNAm sites had a q-value less than 0.05. Additionally, of the 25 regions identified, 12 were associated with neurological diseases or disorders. (4) These findings suggest that there is a neurological component to depression, which should be considered during treatment.

## 1. Introduction

Depression affects over 350 million people globally and is ranked second in worldwide disease burden; it is expected to rank first by 2030 [1,2]. The prevalence of depression peaks in the second and third decades of life and is roughly two times higher in women (24.0%) compared to men (13.3%) in the United States (US), which may be partly due to the willingness of women to report symptoms [1,3,4]. Depression is driven by both social and biological factors, many of which have been poorly elucidated, and largely attributed to differences in reporting bias between sexes [3].

Black women are a group that is at a very high risk of developing depression because they are more likely to be the single head of the household; living in a poorer, higher crime neighborhood; bearing the burden of caring for family members; and dealing with discrimination [5]. Black women are also more likely to be of a lower socioeconomic status [6]. In the US National Survey of American Life, 21.3% of Black women reported depression compared to 10.1% of White women [7]. In addition, the Study of Environment, Lifestyle, and Fibroids (SELF), which focuses on Black women, reported symptoms of depression in 43% of participants [8]. Black women are sometimes unaware of their symptoms of depression due to cultural coping [5]. There is also a stigma related to depression in the Black community, and depression can be seen as a sign of weakness, leading to the reluctance of Black women to report symptoms [5]. Even when diagnosed with depression, Black women have more severe and chronic disease [9].

Depression is characterized by symptoms such as low mood, feelings of worthlessness or hopelessness, anhedonia, appetite changes, sleep disturbances, and suicidal ideation [10]. Women are more likely to report symptoms like weight gain, increase in appetite, and somatic symptoms [3]. Women are also more likely to experience social disadvantages than men [3]. Many hormones impact the risk of depression, including testosterone, estrogen, serotonin, and cortisol [11]. These hormones vary throughout the lifespan and interact with social factors, putting some groups at higher risk than others of developing depression [12]. In addition, the production of these hormones can change in response to stress and affect cognitive and emotional function [11].

Diagnosing depression can be difficult due to the varied symptom presentations and complicated etiology [13]. A formal diagnosis of depression is generally made by a trained mental health professional who identifies symptoms based on the Diagnostic and Statistical Manual of Mental Disorders or the International Classification of Diseases [1]. Individuals may also be screened for depressive symptoms using measurement instruments designed for this purpose, which can be self-administered [1]. However, some people may not have access to a trained professional, or stigma may prevent them from seeking care [14]. Patients may also be reluctant to divulge sensitive information to providers [15]. For these reasons, screening and diagnosis are difficult, and many people remain undiagnosed and untreated [5].

The utilization of genetic data is being explored as a more objective method for diagnosis. Depression has an estimated heritability of approximately 40%, and both genetic and environmental factors likely contribute to depression [2,16]. The complex interplay between societal factors that are known to impact depression—systemic racism, discrimination, and socioeconomic deprivation—interacts with underlying biological factors (e.g., genes) to increase stress, leading to epigenetic changes and ultimately depression [17,18]. DNA methylation is an epigenetic modification that can affect gene expression without altering DNA sequences [19]. These modifications are responsive to environmental stimuli and may be reversible [19]. Psychosocial stressors, such as experiences with discrimination, have been shown to influence DNA methylation, leading to changes in gene expression [13].

Other studies have investigated the association between DNA methylation and depression [19,20]. Differential methylation related to depression has been observed in several genes, including *BDNF*, *NTRK2*, *SLC6A4*, *NR3C1*, *PRIMA1*, and *OXTR*, but results across studies are inconsistent [19,21]. However, no studies have investigated the relationship between DNA methylation and depression in Black women. Therefore, the objective of this study is to examine associations between DNA methylation and depressive symptoms in Black women.

## 2. Results

Individual characteristics are summarized in Table 1. A total of 235 women had usable DNA methylation data and complete responses on the BDI. The average age was 31, and 8% were Hispanic or Latino. Approximately 5% had some high school education, and 36% had a high school diploma or general educational diploma (GED). Over half of the participants (65%) were single. Nearly half (45%) reported an annual household income of less than USD 15,000. Unemployment benefits were received by 23%, 3% received Temporary Assistance for Needy Families (TANF) or Family Investment Program (FIP), and 3% reported no cash income. Over a quarter of participants (28%) reported receiving food stamps, 23% received a housing subsidy, 17% received heating assistance, and 17% received Women, Infants, and Children (WIC) benefits. Only 18% reported always having money for basic things, and 40% responded with “most of the time”. When asked how often they have money for special things, 13% of participants responded with “never”, and 44% responded with “sometimes”. Over half (61%) of participants had Medicaid insurance, and 5% had no insurance. Approximately one-fifth (21%) were not living in permanent housing at the time of the interview. Depressive symptoms were categorized into four levels: normal (*n* = 159), mild to moderate (*n* = 45), moderate to severe (*n* = 18), and severe (*n* = 13). Nearly a third (32%) of participants reported experiencing depressive symptoms at a level higher than normal.

Bivariate associations were examined between participant characteristics and depression symptoms (Table 1). Depressive symptoms were significantly associated with household income, type of cash income, type of non-cash income, whether participants had money for basic things or special things, EOD scores, and RRES scores.

When participants were stratified by severity of depressive symptoms, there was a significant difference in marital status between groups (*p* = 0.003) with a higher percentage of the moderate-to-severe and severe groups reporting single marital status (Table S1). A larger proportion of the severe group reported no cash income and a smaller proportion of this group indicated receiving employment income. Unemployment benefits were received by a larger proportion of the mild-to-moderate, moderate-to-severe, and severe groups. More of the mild-to-moderate group received food stamps. A larger portion of the groups reporting depressive symptoms indicated having money for basic things only sometimes or about half the time; a larger portion of these groups also reported never having money for special things. More of the mild-to-moderate and moderate-to-severe participants reported not living in permanent housing at the time of the interview. The group reporting a severe level of depressive symptoms had significantly higher scores on the EOD and RRES than the normal group.

Differentially methylated CpG sites associated with depressive symptoms were identified using dmpFinder. A total of six CpG sites were identified with a q-value less than 0.05 (Table 2). However, none of these sites were annotated to a gene; all were located in noncoding regions.

Bumphunter identified 25 differentially methylated regions associated with depressive symptoms (Table 3). These regions were annotated to nearby genes and were located inside introns (*n* = 16), inside exons (*n* = 3), downstream (*n* = 1), or upstream from the gene (*n* = 4), or in the promoter region (*n* = 1). The regions inside introns, like the significant CpGs in Table 2, were noncoding. Twelve of the nearby genes were associated with neurological diseases or disorders, including spasticity, neuroblastoma, and neuropathy [22]. Five of the genes were involved in neurological pathways, and two genes had a neurologically related function [22].

## 3. Discussion

In this secondary analysis of data from the InterGEN study, we identified relationships between depressive symptoms and six differentially methylated positions, and twenty-five differentially methylated regions associated with depressive symptoms. In this study, a total of 12 annotated genes were associated with neurological diseases: *GLRX5*, *CLEC1B*, *NBPF8*, *NDUFA10*, *PLEKHM3*, *L3MBTL4*, *ADCY8*, *ARHGEF10*, *TAFA5*, *OTOF*, *TANGO2*, and *LINC02915*. Two of the annotated genes have a direct neurological impact: *CLEC1B* is involved in the process of cognition, and *PKD1L3* functions in the reception of the sense of taste [22].

Glutaredoxin 5 (*GLRX5*) has been associated with neuromuscular spasticity and neuroimaging abnormalities [23]. The *CLEC1B* gene was found to be correlated with cognitive function [24] and bipolar disorder in humans [25]. It has also been associated with bipolar disorder in an analysis of cerebrospinal fluid [25]. Neuroblastoma Breakpoint Family genes, such as *NBPF8*, are associated with neuroblastoma, a cancer that arises from nerve cells, often in the sympathetic nervous system [26].

The *NDUFA10* gene has been linked to Leigh syndrome, a neurogenerative disease characterized by lesions in the basal ganglia and/or brainstem and deficits in motor function [27]. This gene also functions in the biogenesis of mitochondrial complex I; dysfunction in the mitochondria can lead to neurological disorders such as seizures, dystonia, ataxia, optic atrophy, and sensorineural hearing loss [28]. The *PLEKHM3* gene has been associated with neuropathy and chordoma [22].

The L3MBTL4 gene has been linked to N Syndrome, which can cause seizures, nystagmus, spasticity, and severe intellectual impairment, and Bardet–Biedl Syndrome, which often presents with visual and intellectual impairments [29,30]. Adenylate cyclase 8 (*ADCY8*) functions in the regulation of synapse activity and has been associated with dissociative amnesia [22,31]. Rho Guanine Nucleotide Exchange Factor 10 (*ARHGEF10*) has previously been linked to nerve conduction velocity, neuropathy, and elevated risk of ischemic stroke [22,32]. In mice, the *TAFA5* gene was associated with depression and was expressed in brain tissue [33].

The otoferlin gene (*OTOF*) functions in the sensory processing of sound and smell [22]. It has been associated with autosomal recessive deafness and auditory neuropathy [22,34]. *TANGO2* has been linked to neurodegeneration and encephalopathy [18]. Deficiencies in this gene can lead to developmental delays, intellectual disability, spasticity, gait abnormality, speech impairment, and seizures [35]. *LINC02915* is noncoding and located in an intron, but it has been associated with spastic paraplegia [22].

With the exception of *TAFA5*, none of these genes have previously been associated with depression. However, their association with depressive symptoms in this study and the related neurological functions suggest possible links between depression and the neurological system. Depression has previously been classified as both a mental illness and a neurological or neuropsychiatric disorder and is often treated by influencing the levels of neurotransmitters in the brain, such as serotonin, norepinephrine, and dopamine [36,37]. However, as many individuals fail to respond to traditional pharmacological therapies, treatments directly targeting the brain are being implemented, such as transcranial magnetic stimulation, electroconvulsive therapy, and vagus nerve stimulation [37]. In addition, depressive episodes have been induced during high-frequency stimulation of the substantia nigra in patients with no prior psychiatric history, and positron emission tomography performed during a depressive episode showed dysfunctional circuitry between cortical and subcortical regions of the brain [38]. Low self-esteem, one of the common symptoms of depression, has been associated with decreased neural activity in the amygdala in functional magnetic resonance imaging studies [39].

This disorder has also been associated with increased levels of inflammatory cytokines, which can negatively affect pathways associated with mood, emotion, and cognition, such as neurotransmission, activation of microglia, dysregulation of the hypothalamic–pituitary–adrenal axis, and brain plasticity [40]. In a transcriptomic study of depression using RNA-Seq analysis of samples of microglia from the prefrontal cortex, there were significant differences between samples from depressed individuals and healthy controls [41]. In addition, this study found significant changes in signaling pathways related to cell communication, cell adhesion, cell growth, and differentiation [41]. Continued emphasis on the neurological aspects of depression could lead to novel discoveries in the pathology of this disorder, improvements in drug development and repurposing, and advances in precision medicine for individuals with depression.

### Limitations

There were a few limitations to this study. The participants were all healthy with some reporting depressive symptoms during screening. Analyzing the DNAm of a sample of depressed participants could identify additional differential methylation. Depressive symptoms were assessed in the current study using the BDI. Ideally, as many depressive symptom scales exist, multiple scales should be used to screen participants, and their associated genes can be compared for consistency [42].

In addition, sequencing was performed using saliva samples. Prior research has suggested that epigenetic changes associated with depression are more enriched in neuronal cells [19]. The genes associated with depression in previous studies may not have been replicated in this study because previous studies of epigenetics and depression were not conducted on a sample of Black women. A systematic review of 67 DNAm studies focusing on depression included studies performed in the US, Australia, Asia, and Europe, but did not include any studies performed in Africa [20]. In addition, some studies only focused on one or a few candidate genes while the current study used an epigenome-wide approach [19,20]. Many of the included studies reported no associations at all with depression [20].

The different studies did not all recruit study participants with the same depression phenotype; different phenotypes included major depressive disorder, postpartum depression, “depression in general”, and depressive symptoms [20]. Sample types were also inconsistent and included whole blood, buccal cells, leukocytes, brain tissue, saliva and peripheral blood mononuclear cells [20]. These varied results and methods indicate that associations are difficult to replicate across studies. Future research should focus on more diverse populations and include more depressed individuals to compare with healthy controls. It would also be helpful for researchers to establish a gold-standard sample type because different cell types can yield different results [19]. While prior research has suggested that neuronal cells are ideal for showing DNAm changes, this sample type is more difficult to obtain from live participants than from blood or saliva [19]. Future research could also include brain imaging of study participants to pinpoint differences between individuals with depression and healthy controls or to identify depression subtypes [39].

## 4. Materials and Methods

### 4.1. Theoretical Framework

**Figure 1:**
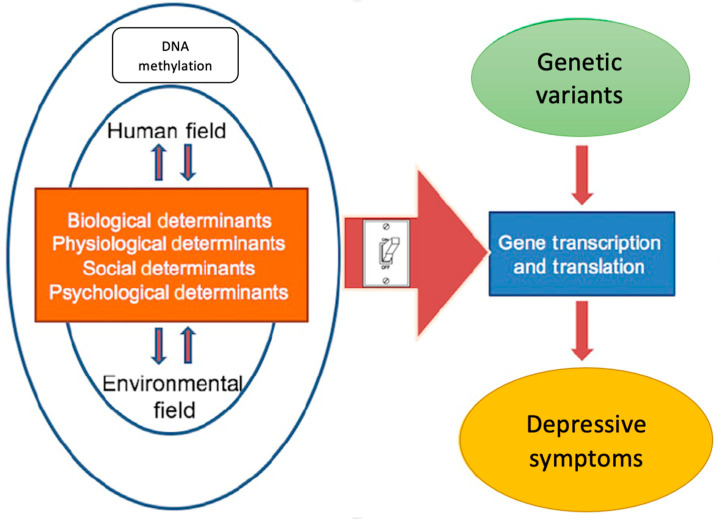
Adapted UIC Model for Genetic and Epigenetic Research.

This study was guided by the University of Illinois at Chicago (UIC) Model for Genetic and Epigenetic Research (Figure 1), which asserts that genetic and environmental influences interact and lead to a phenotype [43].

Humans constantly interact with their environment, and these interactions can affect health status [43]. Biological, physiological, social, and psychological factors can influence DNA methylation and gene expression, leading to depressive symptoms.

### 4.2. Study Design and Sample

We conducted a secondary analysis of data from the Intergenerational Impact of Genetic and Psychological Factors on Blood Pressure (InterGEN) study. This study was a longitudinal study of Black mothers and their children aged 3–5 [44]. The aim was to examine the effects of gene-environment interaction on blood pressure in Black women and their children [44]. Participants were recruited from early childhood education centers and community events in Southwest and Central Connecticut from 2014 to 2019 [44]. Inclusion criteria are women aged 21 or older with a biological child aged 3 to 5 years, who identify as Black or African American, are fluent in English, and have not been diagnosed with a psychiatric or cognitive disorder that could affect the reporting of data [44].

Interviews were conducted at four time points, and information on demographics and social determinants of health and measurements of physical health were collected at each interview [44]. Demographics and information on social determinants of health were obtained in face-to-face interviews using Audio Computer-Assisted Self-Interviewing [44]. In this interviewing method, questions and responses are displayed on the computer screen, and participants can hear the question via audio [44].

### 4.3. Instruments and Measures

#### 4.3.1. Sociodemographic Variables

Sociodemographic variables included age, ethnicity, marital status, income, education, insurance status, and housing. All participants were female and self-identified their race as Black or African American. The categories for marital status were married, single, divorced, separated, or living with but not married to a significant other. Education levels were less than high school, high school diploma or GED, some college, associate’s degree, bachelor’s degree, master’s degree, or doctorate. Health insurance response options were insured or uninsured, and housing options were permanent or nonpermanent housing.

#### 4.3.2. Depressive Symptoms

Symptoms of depression were assessed at each interview using the Beck Depression Inventory (BDI) [45]. This scale contains 21 items, each of which describes a specific symptom of depression: mood, pessimism, feelings of failure, lack of satisfaction, feelings of guilt, sense of punishment, self-hate, self-accusations, self-punitive wishes, crying spells, irritability, social withdrawal, body image issues, work inhibition, sleep disturbances, fatigue, appetite changes, weight changes, somatic preoccupation, and loss of libido [46]. Each item contains four to six ordinal statements meant to reflect the severity of the symptom from neutral to maximum severity [45]. The statements are assigned numerical values from 0 to 3, and the scores for each statement are summed into a total score [45]. The severity of depressive symptoms is classified as follows: <10 is none or minimal; 10–18 is mild to moderate; 19–29 is moderate to severe; and 30–63 is severe [46].

#### 4.3.3. Perceived Discrimination

Two instruments were used to assess perceived discrimination: the Experiences of Discrimination (EOD) and the Race-Related Events Scale (RRES). The EOD consists of an 11-item experience of discrimination subscale and a 9-item major discrimination subscale [47]. The summary score for each subscale is the total number of situations where participants reported experiencing discrimination or receiving unfair treatment [47]. The RRES is a 23-item scale of yes-or-no questions that assess whether certain discriminatory situations have been experienced, such as “being treated rudely or coldly” [48]. The “yes” responses are summed to create a total score [48]. There is an additional open-ended item for participants to fill in situations not listed [48].

#### 4.3.4. DNA Methylation Preprocessing

Participants provided saliva samples using Oragene-500 Format tubes at the first interview [49]. They were asked not to eat, drink, smoke, or chew gum for 30 min prior to providing the sample, and each participant spit into a tube repeatedly until the liquid reached a fill line of two milliliters (mL) [49]. Participants failing to fill the tube to the 2 mL line were given a clear lollipop to stimulate saliva production [49]. Each participant’s tube was then labeled with a barcode, which was entered into a freezer inventory; samples were then stored at 4 °C [49]. The Illumina Infinium Methylation EPIC (850 K) BeadChip was used to analyze epigenome-wide DNAm [49]. DNAm data were obtained through measurement of the fluorescent signals from methylated and unmethylated signals at each site; Intensity Data (IDAT) files were generated to store these measurements [49,50].

The raw IDAT files, along with the manifest file containing phenotype data, were imported into RStudio using the minfi package [51]. In DNAm studies, preprocessing plays a crucial role in ensuring the quality and reliability of the data [52]. The key preprocessing steps include, but are not limited to, the following: (1) probe filtering, (2) background correction, (3) color balance adjustment, (4) data normalization, (5) probe-type bias correction, and (6) batch effect removal [53].

In our initial quality control step, detection *p*-values were calculated using the minfi “detectionP” function. We excluded probes from further analyses if the probes had a detection *p* > 0.01 in more than 20% of the samples [54]. To improve the accuracy of downstream analysis, methylation data were further preprocessed using the minfi “preprocessFunnorm” function [55]. Specifically, functional normalization removes unwanted technical variation using control probes as surrogates [53] and has been shown to outperform several other normalization approaches [56]. The final methylation data included 654,407 probes. The methylation level at each CpG site was quantified as a beta-value, which ranges from 0 (indicating no methylation) to 1 (indicating complete methylation) [57].

#### 4.3.5. Statistical Analysis

The primary outcome measure was the level of depressive symptoms, a variable derived from the BDI scores. Descriptive statistics such as mean and standard deviation for the continuous variables and frequency and percentage for the categorical variables were calculated for the sociodemographic characteristics. Participants with missing data in BDI items were excluded from the analysis.

Differential methylation analysis was performed to identify differentially methylated CpG sites and regions associated with the level of depressive symptoms. Several R packages, including limma, minfi, bumphunter, and MissMethyl were utilized for this analysis. The limma package is widely used for differential expression analysis, but it can be adapted for differential methylation analysis in combination with minfi [58]. The “lmFit” and” eBayes” limma functions were used to test for differential methylation. Differential analyses were conducted at both the region and probe levels. The function “bumphunter” from the minfi package was employed to identify differentially methylated regions (referred to as bumps) associated with depressive symptoms. Additionally, the “dmpFinder” function was utilized to identify differentially methylated positions (CpG sites). Gene-set enrichment analysis was performed using the “gometh” function from missMethyl, as well as the “annotateTranscripts” and “matchGenes” functions from bumphunter to gain insights into the biological significance of the affected genes identified. Analyses for this study were performed in RStudio with R version 4.2.2.

## 5. Conclusions

In the current study, we examined associations between depressive symptoms and DNAm among Black women in the InterGEN study. Black women are an understudied group in epigenomic research [49], and this study adds to the literature on differential methylation and depressive symptoms in this population. Differential methylation analysis identified six CpG sites and 25 differentially methylated regions associated with levels of depressive symptoms, and half of those regions were annotated to genes associated with neurological pathways, functions, diseases, or disorders. This suggests that there is a neurological component to depression [36], which should be considered by clinicians when treating individuals with depression. In addition, neurological pathways could possibly be targets for new drugs developed to treat depression. Further research is necessary to determine whether these findings can be replicated in a more diverse population or whether these findings are specific to Black women. Subsequent studies should also strive for consistency in sample types and depressive-symptom screening methods to eliminate variability due to the use of different methods.

## Figures and Tables

**Table 1:** Participant Characteristics.

Variable Category (*N* = 235)	Variable Level	*N* (%)	*p*-Value
Age (mean (SD))		31.31 (5.77)	0.40
Ethnicity	Not Hispanic or Latino	215 (91.5)	0.89
	Hispanic or Latino	19 (8.1)	
Education Level	Less than high school	12 (5.1)	0.32
	High school diploma or GED	84 (35.7)	
	Some college, no degree	79 (33.6)	
	Associate’s degree	26 (11.1)	
	Bachelor’s degree	25 (10.6)	
	Graduate degree	9 (3.9)	
Marital Status	Single	153 (65.1)	0.55
	Living with but not married	11 (4.7)	
	Married	57 (24.3)	
	Separated	3 (1.3)	
	Divorced	11 (4.7)	
Household Income	Less than USD 15,000	107 (45.3)	<0.001
	USD 15,000 to USD 34,999	69 (29.4)	
	USD 35,000 to USD 49,999	28 (11.9)	
	USD 50,000 to USD 74,999	13 (5.5)	
	USD 75,000 to USD 99,999	7 (3.0)	
	USD 100,000 or higher	4 (1.7)	
Cash Income	Employment earnings	161 (68.5)	<0.001
	Unemployment benefits	55 (23.4)	
	TANF/FIP	6 (2.6)	
	No cash income	8 (3.4)	
Non-cash Income	Food stamps	66 (28.1)	<0.001
	Housing subsidy	56 (23.8)	
	Heating assistance	41 (17.4)	
	WIC	18 (17.7)	
	No non-cash income	4 (1.7)	
Money for Basic Things	Never	6 (2.6)	0.01
	Sometimes	49 (20.9)	
	About half the time	41 (17.4)	
	Most of the time	95 (40.4)	
	Always	42 (17.9)	
Money for Special Things	Never	32 (13.6)	0.008
	Sometimes	104 (44.3)	
	About half the time	37 (15.7)	
	Most of the time	47 (20.0)	
	Always	13 (5.5)	
Health Insurance	Yes	222 (94.5)	0.69
	No	13 (5.5)	
Insurance Type	Private/Employer-provided	34 (14.5)	0.79
	Government-provided	36 (15.3)	
	Medicaid	145 (61.7)	
	Other	7 (3.0)	
Permanent Housing	Yes	183 (77.9)	0.14
	No	50 (21.3)	
Experiences of Discrimination (mean (SD))		3.90 (8.08)	<0.001
Race-Related Events Scale (mean (SD))		3.62 (4.67)	<0.001
Beck Depression Inventory	None or minimal	159 (67.7)	
	Mild to moderate	45 (19.1)	
	Moderate to severe	18 (7.7)	
	Severe	13 (5.5)	

**Table 2:** Significant CpG Sites Associated with Level of Depressive Symptoms.

CpG	*p*-Value	Q-Value
cg23107033	1.46 × 10^−8^	0.01143
cg17212404	2.64 × 10^−8^	0.01143
cg15085109	4.84 × 10^−8^	0.012599
cg01954746	5.82 × 10^−8^	0.012599
cg24468104	1.62 × 10^−7^	0.027984
cg04636364	2.47 × 10^−7^	0.035598

**Table 3:** Annotation of Differentially Methylated Regions Associated with Level of Depressive Symptoms.

Gene Symbol	Gene Name	Region	Related Pathways	Annotation/Function	Associated Diseases/Disorders
*GLRX5*	Glutaredoxin 5	Downstream	Mitochondrial iron-sulfur cluster biogenesis, p21-activated protein kinase (PAK) pathway ^1^	Electron transfer activity, 2 iron-2 sulfur cluster binding	Anemia-Sideroblastic-Pyridoxine-Refractory, Spasticity-Childhood-Onset with Hyperglycinemia ^3^
*OSBPL9*	Oxysterol Binding Protein Like 9	Inside exon	Synthesis of bile acids and salts, metabolism	Lipid binding	Lenz–Majewski Hyperostotic Dwarfism
*ADAMTS17*	ADAM Metallopeptidase with Thrombospondin Type 1 Motif 17	Inside intron	O-linked glycosylation of mucins, metabolism of proteins	Peptidase activity, metalloendopeptidase activity	Weill–Marchesani Syndrome 4, Anterior Segment Dysgenesis
*CLEC1B*	C-Type Lectin Domain Family 1 Member B	Inside intron	Cellular responses to stimuli and elevated platelet cytosolic Ca^2+^	Transmembrane signaling receptor activity, carbohydrate binding, cognition ^2^	Bleeding disorder—Platelet-Type 11, Bladder Squamous Cell Carcinoma, Bipolar disorder ^3^
*NBPF8*	Neuroblastoma Breakpoint Family Member 8	Inside intron	None	None	Neuroblastoma ^3^
*NDUFA10*	NADH: Ubiquinone Oxidoreductase Subunit A10	Promoter	Respiratory electron transport, ATP synthesis by chemiosmotic coupling, heat production by uncoupling proteins, Complex I biogenesis ^1^	NADH dehydrogenase activity, nucleoside kinase activity	Mitochondrial Complex I Deficiency—Nuclear Type 22 ^3^, Leigh Syndrome ^3^
*SULF2*	Sulfatase 2	Inside exon	None	Calcium ion binding, arylsulfatase activity	Inflammatory Bowel Disease
*SH3BP2*	SH3 Domain Binding Protein 2	Inside intron	TCR signaling in naïve CD4+ T cells	SH3 domain binding, obsolete SH3/SH2 activity	Cherubism, Giant Cell Reparative Granuloma
*SLC19A1*	Solute Carrier Family 19 Member 1	Upstream	Metabolism of water-soluble vitamins and cofactors, methotrexate pathway—pharmacokinetics	Oxidoreductase activity, folic acid transmembrane transporter activity	Megaloblastic Anemia—Folate-Responsive, Immunodeficiency 114—Folate-Responsive
*PLEKHM3*	Pleckstrin Homology Domain Containing M3	Upstream	None	Myoblast differentiation	Median Neuropathy ^3^, Chondroid Chordoma ^3^
*CTNNBL1*	Catenin Beta Like 1	Inside intron	Processing of Capped Intron-Containing Pre-mRNA	Binding, enzyme binding	Immunodeficiency 99 With Hypogammaglobulinemia and Autoimmune Cytopenias, Immunodeficiency With Hyper-Igm—Type 2
*L3MBTL4*	L3MBTL Histone Methyl-Lysine Binding Protein 4	Inside intron	None		
*ADCY8*	Adenylate Cyclase 8	Inside intron	Adora2b-mediated anti-inflammatory cytokine production, beta-2 adrenergic-dependent CFTR expression	Nucleotide binding, adenylate cyclase activity	Dissociative Amnesia ^3^, Precocious Puberty—Central 1
*LOC574538*	Uncharacterized LOC574538	Inside intron	None	None	None
*ZZEF1*	Zinc Finger ZZ-type and EF-Hand Domain Containing 1	Inside intron	None	Calcium ion binding	None
*L3HYPDH*	Trans-L-3 Hydroxyproline Dehydratase	Inside intron	None	Hydro-lyase activity, trans-L-3-hydroxyproline dehydratase activity	None
*LGALS14*	Galectin 14	Upstream	None	Carbohydrate binding, inducer of T-cell apoptosis	None
*ARHGEF10*	Rho Guanine Nucleotide Exchange Factor 10	Inside exon	p75 NTR receptor-mediated signaling ^1^, GPCR pathway	Guanyl-nucleotide exchange factor activity, kinesin binding	Slowed Nerve Conduction Velocity ^3^, Autosomal Dominant and Axonal Neuropathy ^3^
*TAFA5*	TAFA Chemokine Like Family Member 5	Inside intron	None	Regulation of cell proliferation and migration	None
*PKP2*	Plakophilin 2	Inside intron	Keratinization, nervous system development ^1^	Binding, protein kinase C binding	Arrhythmogenic Right Ventricular Dysplasia Familial 9, Arrhythmogenic Right Ventricular Cardiomyopathy
*OTOF*	Otoferlin	Inside intron	Sensory processing of sound ^1^, olfactory signaling pathway ^1^	Calcium ion binding, AP-2 adaptor complex binding	Deafness—Autosomal Recessive 9 ^3^, Arthrogryposis and Ectodermal Dysplasia
*TANGO2*	Transport and Golgi Organization 2 Homolog	Inside intron	22q11.2 copy number variation syndrome	None	Metabolic Crises—Recurrent—with Rhabdomyolysis, Cardiac Arrhythmias and Neurodegeneration ^3^, Tango2-Related Metabolic Encephalopathy and Arrythmias ^3^
*LINC02915*	Long Intergenic Non-Protein Coding RNA 2915	Upstream	None	None	Spastic Paraplegia 11—Autosomal Recessive ^3^
*PKD1L3*	Polycystin 1-Like 3, Transient Receptor Potential Channel Interacting	Inside intron	None	Taste reception ^2^	Polycystic Kidney Disease

^1^ Neurological pathway. ^2^ Neurologically related function. ^3^ Neurological disease or disorder.

## Data Availability

Data are available from the authors with a Data Use Agreement upon reasonable request.

## Supplementary Materials

The following supporting information can be downloaded at: https://www.mdpi.com/article/10.3390/ijms25147681/s1.
