# Supplementary material for: Epigenome-Wide Association Study of Depressive Symptoms in Black Women in the InterGEN Study
Source: Int J Mol Sci. 2024 Jul 12;25(14):7681. doi: 10.3390/ijms25147681 (PMC11277114; doi:10.3390/ijms25147681)
Supplement: Supplementary file 1 [file ijms-25-07681-s001.zip › ijms-3049073-supplementary.pdf]

Table S1. Participant Characteristics.

Variable Category	Variable Level	Normal (n=159)	Mild to Moderate (n=45)	Moderate to Severe (n=18)	Severe (n=13)	P-value
Age (mean(SD))		31.53 (5.49)	31.64 (6.75)	29.94 (4.80)	29.31 (6.65)	0.40
Ethnicity	Not Hispanic or Latino	144 (90.6)	44 (97.8)	16 (88.9)	12 (92.3)	0.34
	Hispanic or Latino	15 (9.4)	1 (2.2)	2 (11.1)	1 (7.7)	
Education Level	Less than high school	8 (5.0)	3 (6.7)	0 (0.0)	1 (7.7)	0.83
	High school diploma or GED	54 (34.0)	14 (31.1)	10 (55.6)	6 (46.2)	
	Some college, no degree	56 (35.2)	13 (28.9)	6 (33.3)	4 (30.8)	
	Associate's degree	17 (10.7)	7 (15.6)	1 (5.6)	1 (7.7)	
	Bachelor's degree	16 (10.1)	7 (15.6)	1 (5.6)	1 (7.7)	
	Graduate degree	8 (5.0)	1 (2.2)	0 (0.0)	0 (0.0)	
Marital Status	Single	102 (64.2)	24 (53.3)	16 (88.9)	11 (84.6)	0.003
	Married	45 (28.3)	11 (24.4)	0 (0.0)	1 (7.7)	
	Other	12 (7.5)	4 (8.9)	4 (22.2)	1 (7.7)	
Household Income	Less than $15,000	72 (45.2)	20 (44.4)	11 (61.1)	4 (30.8)	0.10
	$15,000 to $34,999	49 (30.7)	10 (22.3)	6 (33.4)	4 (30.8)	
	$35,000 to $49,999	17 (10.7)	8 (17.8)	1 (5.6)	2 (15.4)	
	$50,000 to $74,999	9 (5.7)	4 (8.9)	0 (0.0)	0 (0.0)	
	$75,000 to $99,999	5 (3.1)	2 (4.4)	0 (0.0)	0 (0.0)	
	$100,000 or higher	3 (1.9)	1 (2.2)	0 (0.0)	0 (0.0)	
Cash Income	Employment earnings	113 (71.1)	33 (73.3)	12 (66.7)	3 (23.1)	<0.001
	Unemployment benefits	34 (21.4)	12 (26.7)	4 (22.2)	5 (38.5)	
	TANF/FIP	5 (3.1)	0 (0.0)	1 (5.6)	0 (0.0)	
	No cash income	5 (3.1)	0 (0.0)	1 (5.6)	2 (15.4)	
Non-Cash Income	Food stamps	40 (25.2)	20 (44.4)	3 (16.7)	3 (23.1)	0.003
	Housing subsidy	42 (26.4)	8 (17.8)	3 (16.7)	3 (23.1)	
	Heating assistance	32 (20.1)	6 (13.3)	2 (11.1)	1 (7.7)	
	WIC	13(8.2)	2 (4.4)	2 (11.1)	1 (7.7)	
Money for Basic Things	Never	4 (2.5)	1 (2.2)	1 (5.6)	0 (0.0)	<0.001
	Sometimes	30 (18.9)	9 (20.0)	7 (38.9)	3 (23.1)	
	About half the time	24 (15.1)	8 (17.8)	6 (33.3)	3 (23.1)	
	Most of the time	68 (42.8)	19 (42.2)	4 (22.2)	4 (30.8)	
	Always	33 (20.8)	8 (17.8)	0 (0.0)	1 (7.7)	
Money for Special Things	Never	15 (9.4)	9 (20.0)	6 (33.3)	2 (15.4)	<0.001
	Sometimes	69 (43.4)	21 (46.7)	9 (50.0)	5 (38.5)	
	About half the time	29 (18.2)	2 (4.4)	3 (16.7)	3 (23.1)	
	Most of the time	34 (21.4)	12 (26.7)	0 (0.0)	1 (7.7)	
	Always	12 (7.5)	1 (2.2)	0 (0.0)	0 (0.0)	
Health Insurance	Yes	152 (95.6)	41 (91.1)	17 (94.4)	12 (92.3)	0.69
	No	7 (4.4)	4 (8.9)	1 (5.6)	1 (7.7)	
Insurance Type	Private/Employer-provided	21 (13.2)	2 (11.1)	21 (13.2)	2 (15.4)	0.40
	Government-provided	26 (16.4)	4 (8.9)	2 (22.2)	2 (15.4)	
	Medicaid	101 (63.5)	27 (60.0)	11 (61.1)	6 (46.2)	
	Other	4 (2.5)	1 (2.2)	0 (0.0)	2 (15.4)	
Permanent Housing	Yes	127 (81.1)	34 (75.6)	11 (61.1)	11 (84.6)	0.04
	No	30 (18.9)	11 (24.4)	7 (38.9)	2 (15.4)	
Experiences of Discrimination (mean(SD))		2.60 (4.25)	5.22 (6.68)	2.17 (2.26)	17.62 (25.05)	<0.001
Race-Related Events Scale (mean(SD))		2.78 (4.12)	5.82 (5.23)	4.39 (4.12)	5.15 (6.84)	0.001
